# Epigenetic Mechanisms in Hematologic Aging and Premalignant Conditions

**DOI:** 10.3390/epigenomes7040032

**Published:** 2023-12-12

**Authors:** Bowen Yan, Qingchen Yuan, Olga A. Guryanova

**Affiliations:** Department of Pharmacology and Therapeutics, College of Medicine, University of Florida, Gainesville, FL 32610, USA; qingchenyuan@ufl.edu

**Keywords:** epigenetics, hematopoietic stem cells, aging

## Abstract

Hematopoietic stem cells (HSCs) are essential for maintaining overall health by continuously generating blood cells throughout an individual’s lifespan. However, as individuals age, the hematopoietic system undergoes significant functional decline, rendering them more susceptible to age-related diseases. Growing research evidence has highlighted the critical role of epigenetic regulation in this age-associated decline. This review aims to provide an overview of the diverse epigenetic mechanisms involved in the regulation of normal HSCs during the aging process and their implications in aging-related diseases. Understanding the intricate interplay of epigenetic mechanisms that contribute to aging-related changes in the hematopoietic system holds great potential for the development of innovative strategies to delay the aging process. In fact, interventions targeting epigenetic modifications have shown promising outcomes in alleviating aging-related phenotypes and extending lifespan in various animal models. Small molecule-based therapies and reprogramming strategies enabling epigenetic rejuvenation have emerged as effective approaches for ameliorating or even reversing aging-related conditions. By acquiring a deeper understanding of these epigenetic mechanisms, it is anticipated that interventions can be devised to prevent or mitigate the rates of hematologic aging and associated diseases later in life. Ultimately, these advancements have the potential to improve overall health and enhance the quality of life in aging individuals.

## 1. Introduction

Hematopoietic stem cells (HSCs) play a vital role in maintaining a balanced production of all blood cells throughout a lifetime. However, with advancing age, the regenerative capacity and self-renewal potential of HSCs progressively decline while the likelihood of cellular dysfunction significantly rises. This age-related decline in HSC function is accompanied by various molecular and functional changes. One notable change observed in aged HSCs is a compromised self-renewal and differentiation potential, leading to a skewed production of myeloid cells, a decreased output of red blood cells, and a reduced generation of immune cells. These alterations contribute to conditions such as anemia, increased susceptibility to infections, and a higher risk of developing hematopoietic malignancies [1].

In aged HSCs, researchers have identified global epigenetic changes that occur with aging. These changes can either arise randomly through epigenetic drift or result from somatic mutations in genes encoding epigenetic regulatory proteins [2]. Mutations in loci associated with epigenetic modifiers are frequently observed in patients with hematological malignancies, as well as in healthy elderly individuals who are at risk of developing these conditions [3].

Interventions targeting the aberrant epigenetic program in dysfunctional aged HSCs hold promise for promoting normal hematopoiesis and treating age-related hematopoietic diseases. In recent years, our understanding of epigenetic regulation has expanded significantly, and drugs that target epigenetic modifications have become increasingly integrated into treatment protocols. The reversible nature of epigenetic modifications makes them attractive targets for novel therapeutics. Rejuvenating methods that can reprogram the epigenetic status of aged HSCs or senolytic drugs that selectively deplete senescent cells offer promising translational avenues for attenuating hematopoietic aging [4].

This review aims to provide an overview of the epigenetic changes that occur in aging HSCs and age-related premalignant conditions. Additionally, we will discuss the potential of epigenetic therapeutic interventions in these contexts.

## 2. Epigenetic Regulation of HSCs

Hematopoiesis, the process of blood cell formation, is tightly regulated and relies on the precise epigenetic control of gene transcription. This epigenetic regulation plays a critical role in maintaining the delicate balance between the self-renewal of HSCs and their proper differentiation into various mature blood cell lineages. Epigenetic modifications, such as DNA methylation, histone modifications, and non-coding RNAs, dynamically modulate gene expression patterns, ensuring the appropriate development and function of hematopoietic cells. These epigenetic mechanisms act as guardians, tightly regulating the fate decisions of HSCs and ensuring the generation of diverse and fully functional blood cell populations [3].

### 2.1. DNA Methylation

DNA methylation, an essential epigenetic modification, is primarily associated with gene repression. It is mediated by a family of DNA methyltransferase enzymes, including DNMT1, DNMT3A, and DNMT3B. DNMT1 plays a crucial role in maintaining pre-existing DNA methylation patterns by recognizing and copying the methylation marks from the parental template strand to the daughter strand [5]. On the other hand, DNMT3A and DNMT3B function as de novo DNA methyltransferases, responsible for establishing new DNA methylation patterns during development and stem cell differentiation [6,7]. Additionally, DNMT2, as an RNA methyltransferase, has distinct functions in RNA modification rather than DNA methylation [8].

DNMT1, an essential DNA methyltransferase, plays a critical role in the self-renewal of HSCs. HSCs lacking DNMT1 exhibit defects in self-renewal capacity, as well as impaired homing to the bone marrow (BM) niche and niche retention. The loss of DNMT1 also exerts specific effects on myeloid progenitor cells, leading to enhanced cell cycling and the inappropriate expression of genes associated with mature lineages [5].

The expression of *Dnmt3a* is significantly higher in the long-term hematopoietic stem cells (LT-HSCs) compared to progenitor cells and differentiated cells [6]. The loss of *Dnmt3a* in HSCs impairs differentiation and leads to an expansion of stem cells [6,9]. In contrast, the ablation of Dnmt3b alone in HSCs shows only minor differences compared to wild-type cells. However, when *Dnmt3a* and *Dnmt3b* are both ablated (*Dnmt3a^−/−^Dnmt3b*^−/−^ HSCs), there is a pronounced exacerbation of stem cell expansion and an almost complete blockade of differentiation [10].

The Ten-eleven translocation (Tet) methylcytosine dioxygenases are enzymes that catalyze the conversion of DNA 5-methylcytosine (5 mC) to 5-hydroxymethylcytosine (5 hmC), a key step in DNA demethylation. The knockout of *Tet2* has been shown to enhance the self-renewal and proliferation of HSCs, disrupting both early and late stages of hematopoiesis, including myeloid and lymphoid differentiation. Additionally, *Tet2* knockout has been associated with an increased risk of developing myeloid malignancies [11,12].

Isocitrate dehydrogenase IDH1 (cytosolic protein) and IDH2 (mitochondrial protein) are metabolic enzymes that catalyze the oxidative decarboxylation of isocitrate to produce the α-ketoglutarate (αKG). αKG is essential for the oxidation of 5 mC by TET enzymes. However, IDH1/2 hotspot mutations result in a gain of function in the enzymatic activity, leading to an increased synthesis and accumulation of (R)-2-hydroxyglutarate (2HG) [13]. Moreover, 2HG competitively inhibits αKG-dependent dioxygenases such as TET enzymes, resulting in loss of 5 hmC, gain of 5 mC, and ultimately gain of DNA methylation [14,15].

Collectively, the acquisition of mutations in genes involved in the regulation of DNA methylation, such as DNMT3A, TET2, and IDH1/2, in HSCs leads to enhanced self-renewal and clonal expansion to a pre-leukemia population [15].

### 2.2. Histone Acetylation

Histone acetylation is regulated by histone acetyltransferases (HATs) and histone deacetylases (HDACs), which play key roles in normal and malignant hematopoiesis.

The MYST family of HATs in humans consists of five members: Tip60, MOZ (KAT6A), MORF (KAT6B), HBO1, and MOF (KAT8) [16]. Among these, KAT6A, KAT6B, and KAT8 play crucial roles in hematopoiesis by catalyzing acetylation on specific lysine residues of histone proteins. KAT6A is responsible for H3K9ac, KAT6B catalyzes H3K23ac/H3K14ac, and KAT8 catalyzes H4K16ac on the regulatory regions of target genes [17]. The activity of these HATs is vital for the generation and development of hematopoietic stem and progenitor cells (HSPCs). In particular, MOZ-mediated acetylation has been found to regulate the delicate balance between differentiation and proliferation in normal hematopoiesis [18,19]. MORF, highly expressed in LT-HSCs, shows decreased expression at both the transcript and protein levels with aging. The knockdown of MORF in young LT-HSCs leads to a biased production of myeloid cells over erythroid cells, both in vitro and in vivo [17], a lineage disbalance characteristic of aging hematopoiesis. Similarly, MOF plays a critical role in maintaining hematopoietic stem cells and the engraftment capacity of adult HSCs, particularly in adult hematopoiesis [20]. Interestingly, previous studies have demonstrated that genetic inactivation of any of these histone acetyltransferases leads to HSC exhaustion in mice mimicking aging-related changes, as observed in prior investigations [17,18,19,20,21,22].

Histone acetyltransferase CREB-binding protein (CBP) and its homolog p300 mediate the deposition of the activating H3K27ac histone marks at gene promoters and enhancers. These transcription co-activators play essential but distinct roles in maintaining normal hematopoiesis. A full dose of CBP, but not p300, is crucial for HSC quiescence and self-renewal [23]. p300, but not CBP, is essential for proper hematopoietic differentiation and proliferation [24]. In mice, both CBP and p300 are required for preventing hematologic tumorigenesis [25]. Man et al. show the loss of p300 enhanced the proliferation and self-renewal capacity of *Tet2*-deficient HSPCs, leading to an increased HSPC pool and leukemogenicity in primary and transplantation mouse models [26].

Histone deacetylases (HDACs) are a class of enzymes that regulate gene expression by deacetylation of lysine residues on histone and nonhistone proteins. In normal hematopoiesis, HDACs are widely involved in the development of various lineages [27]. There are several classes of HDACs, including class I (HDAC1, HDAC2, HDAC3, and HDAC8), class IIa (HDAC4, HDAC5, HDAC7, and HDAC9), class IIb (HDAC6 and HDAC10), class III (sirtuins), and class IV (HDAC11) [28].

HDAC1 and HDAC2 are essential regulators of HSC formation and homeostasis. The ablation of HDAC1 and HDAC2 leads to the depletion of HSCs [27,29]. Additionally, the knockdown of HDAC1 using small interfering RNA promotes myeloid differentiation. Conversely, the overexpression of HDAC1 in hematopoietic progenitor cells blocks myeloid but not erythro-megakaryocytic differentiation in mice [30]. HDAC3 serves as a negative regulator in the expansion of normal human HSCs [31]. In mice, the conditional deletion of HDAC3 has been shown to increase the population of stem cells and early progenitor cells while impeding the progression toward lymphoid-primed multipotential progenitor (LMPP) cells and lymphoid lineages [32]. HDAC8 is most highly expressed in the LT-HSC population within the adult hematopoietic hierarchy and plays a critical role in the maintenance of LT-HSC self-renewal. HDAC8-deficient hematopoietic progenitors are compromised in colony-forming cell serial replating capability in vitro and exhibit reduced long-term serial repopulating activity in vivo [33]. SIRT6, a member of the class III histone deacetylases, plays a crucial role in maintaining HSC homeostasis by supporting self-renewal while restraining proliferation. It achieves this by interacting with TCF/LEF1 and inhibiting the transcription of Wnt target genes through the deacetylation of H3K56ac [34].

### 2.3. Histone Methylation

Polycomb repressor complexes 1 and 2 (PRC1 and PRC2) are multi-protein complexes involved in the epigenetic regulation of gene repression. PRC2 is responsible for the trimethylation of histone H3 at lysine 27 (H3K27me3), which is associated with gene silencing. The core components of PRC2 include EZH1/2, EED, and SUZ12. EZH1/2, a histone methyltransferase, catalyzes the addition of methyl groups to H3K27. PRC1 acts downstream of PRC2 and is involved in recognizing the H3K27me3 mark. It further modifies chromatin by adding further modifications, such as monoubiquitination of histone H2A at lysine 119 (H2AK119ub1). PRC1 components include BMI1, RING1A/RING1B, and CBX proteins [35]. Mice with deletions of the key components of PRC1 or PRC2 complex, such as Bmi [36], Ezh1 [37], Ezh2 [38], and Suz12 [39], experience HSC exhaustion. PRC2 function can also be disrupted by inactivating somatic mutations in *ASXL1* (Additional Sex Combs-Like 1)*,* which results in the loss of PRC2-mediated H3K27me3 at target loci, leading to myeloid transformation [40].

Demethylation of H3K27 is regulated by two distinct enzymes: ubiquitously transcribed tetratricopeptide repeat, chromosome X (UTX), also known as KDM6A, and Jumonji-C (JmjC) domain-containing protein-3 (JMJD3), also known as KDM6B. These enzymes specifically recognize and bind to histone mark H3K27me3. Demethylation of H3K27 by KDM6A or JMJD3 results in the conversion of repressive chromatin to a more permissive state. This allows for the recruitment of coactivators and other chromatin-modifying enzymes that promote gene activation [41,42]. KDM6A played an essential role in the hematopoiesis stem cell homing and engraftment [43,44]. The lack of KDM6A is most detrimental to female hematopoiesis due to the partial redundancy with its Y chromosome paralog UTY [45]. The histone demethylase KDM6B is upregulated in a wide range of blood disorders. It is necessary for HSC self-renewal in response to inflammatory and proliferative stress. Mallaney et al. have shown that a loss of *Kdm6b* leads to the depletion of phenotypic and functional HSCs in adult mice [46].

H3K4 methyltransferases include MLL (Mixed-Lineage Leukemia) proteins and SET family proteins [47]. *MLL1*, but not *MLL2*, is frequently involved in chromosomal rearrangements and the formation of fusion proteins that drive leukemia. *Mll1* is required to produce functional HSCs in embryo development [48]. The knockdown *of Mll3* or knockout of *Mll4* in HSPCs results in impaired differentiation of HSPCs and increased cell expansion [49,50]. The deletion of *Setd1a* in bone marrow hematopoietic cells blocked B cell differentiation from the pro-B to pre-B cell stage [51].

The H3K4 demethylases KDM5B (Jarid1b) and KDM1A (lysine-specific demethylase 1, LSD1) also play essential roles in the regulation of HSC function. *Jarid1b* is highly expressed in primitive hematopoietic compartments and is overexpressed in acute myeloid leukemias (AML). The deletion of *Jarid1b* compromises the HSC self-renewal capacity in mice [52]. On the other hand, LSD1 plays a critical role in repressing HSPC gene expression during hematopoietic differentiation. The loss of LSD1 results in the incomplete silencing of HSPC genes, leading to compromised differentiation [53].

H3K9me3 is a histone modification associated with transcriptional repression and the formation of heterochromatin. Several H3K9 methyltransferases, including SUV39H1, SUV39H2, G9a (EHMT2), GLP (EHMT1), and SETDB1, contribute to the establishment of H3K9 methylation marks [54]. During stem cell differentiation, global chromatin rearrangements occur, and the formation of heterochromatin by H3K9 methylation plays a role in regulating HSC differentiation. The inhibition of the histone methyltransferase G9a, which prevents heterochromatin formation, has been shown to delay HSC differentiation [55]. SUV39H1 and SUV39H2 are involved in the maintenance of HSPC functions. The loss of SUV39H function in mice has been shown to disrupt normal hematopoiesis and impair B lymphoid differentiation [56]. In addition, SETDB1 is required for stem cell maintenance by repressing genes associated with non-hematopoietic cell lineages. Mice with HSC-specific *Setdb1* deficiency experience rapid loss of HSCs and progenitor cells from the bone marrow [57,58].

### 2.4. Noncoding RNAs

Non-coding RNAs (ncRNAs) play a crucial role in the regulation of HSC function. These RNA molecules, which do not encode proteins, have been found to participate in diverse cellular processes, including the maintenance, self-renewal, and differentiation of HSCs.

MicroRNAs (miRNAs) are a class of small ncRNAs that regulate gene expression by post-transcriptionally inhibiting translation or by stimulating target mRNA cleavage [59]. In HSCs, specific miRNAs have been identified as key regulators of HSC self-renewal and differentiation, including miR-22, miR-29a, miR-125, miR-126, and the miR-132/122 cluster [59]. For example, miR-125a and miR-125b have been shown to promote HSC self-renewal and survival and to block further differentiation [60,61,62].

Long non-coding RNAs (lncRNAs) have also emerged as important regulators of HSC function. These longer ncRNAs can modulate gene expression at multiple levels, including transcriptional and post-transcriptional regulation. LncRNAs can act as molecular scaffolds, interacting with chromatin-modifying complexes and influencing the epigenetic state of genes involved in HSC regulation [63,64]. For example, lncRNA *Spehd* has been shown to regulate HSPCs and is required for multilineage differentiation [65].

Overall, the dysregulation of ncRNAs can have profound effects on HSC function and contribute to hematopoietic disorders. Understanding the roles of ncRNAs in HSC regulation provides insights into the molecular mechanisms underlying hematopoiesis and offers potential therapeutic targets for the treatment of hematopoietic diseases.

### 2.5. Interplay of Epigenetic Regulators

In general, there is a complex interplay between DNA methylation, histone modifications, and noncoding RNAs in regulating HSC functions. These different layers of epigenetic modifications are often interconnected, with changes in histone modifications being accompanied by alterations in DNA methylation. For example, some of the gene-silencing activities of DNMT3A are intimately tied to chromatin modifications through interactions with specific proteins. DNMT3A can interact with histone modifiers involved in gene repression, such as SUV39H1, SETDB1, and G9A [66,67,68,69], which are linked to H3K9 methylation. Furthermore, DNMT3A plays a role in coordinating splicing through its recruitment of the core spliceosome protein SF3B1 to RNA polymerase and mRNA, contributing to HSC activation [70]. Additionally, the interaction of EZH2 with DNMT3A, DNMT3B, and DNMT1 is required for the DNA methylation of EZH2 target promoters [10,71,72]. Studies have provided evidence indicating that the microRNA miR-125b acts as a negative regulator of histone methyltransferase SUV39H1, leading to a widespread decrease in the levels of H3K9me3 [56].

## 3. Alterations to the Epigenome in HSCs Aging

### 3.1. Imbalance of Histone Modifications

Aging is accompanied by significant alterations in all layers of epigenetic regulation in HSCs. These shifts are particularly evident in the patterns of histone modifications, which are pivotal for gene expression regulation and chromatin structure organization. The age-related modifications and dysregulation of the epigenetic machinery have profound implications for the functional characteristics of HSCs and can significantly impact the overall process of hematopoiesis during aging.

Sun et al. showed activating H3K4me3 levels increase in old HSCs with broader peaks, especially over genes regulating self-renewal and HSC identity. Additionally, repressive H3K27me3 peaks also broaden with age [73]. The expression of the histone methyltransferase SUV39H1/KMT1A decreases with age in both human and mouse HSCs, resulting in a global reduction in the H3K9me3, disruption of heterochromatin function, and perturbation of B lymphoid differentiation [56,74]. Adelman et al. demonstrated that during normal human aging, HSCs undergo age-associated reprogramming, marked by a redistribution of DNA methylation and reductions in histone marks such as H3K27ac and H3K4me1. This epigenetic reprogramming in aged HSCs significantly affects developmental and cancer pathways, consequently elevating susceptibility to leukemia [74]. Furthermore, levels of microRNA miR-125b, which negatively regulates the expression of histone methyltransferase SUV39H1, increase in human HSCs with age [56]. Studies have shown inhibiting miR-125b and upregulating SUV39H1 in aged HSCs boosts their B cell output [56]. The H3K27me3 demethylase UTX also played an essential role in maintaining a youthful phenotype in HSCs. Thus, UTX-deficient HSCs acquire an early aging phenotype characterized by myeloid skewing, impaired hematopoietic reconstitution, and increased susceptibility to leukemia [44]. In addition, the histone acetyltransferase KAT6B, whose expression declines with age, is important for maintaining a youthful HSC differentiation profile. Knockdown of *Kat6b* in LT-HSCs promotes the expression of aging-associated genes [17]. Class III histone deacetylases (HDACs), such as SIRT1 and SIRT3, play a crucial role in preserving the youthful characteristics of HSCs and protecting them from aging-related decline. SIRT1 functions by facilitating the nuclear localization and activation of FOXO3, a key transcription factor involved in HSC homeostasis [75]. Moreover, SIRT1 negatively regulates the mTOR signaling pathway, which is associated with aging and cellular senescence [76]. On the other hand, SIRT3 is involved in maintaining the acetylation landscape of mitochondrial proteins within stem cells. The age-related downregulation of SIRT3 leads to altered acetylation patterns and increased production of reactive oxygen species (ROS), contributing to HSC aging. Interestingly, the upregulation of SIRT3 has shown the potential to rescue functional deficiencies in aged HSCs, offering a promising avenue for rejuvenation strategies [77,78]. Collectively, these alterations in histone marks and epigenetic regulators enhance the self-renewal capacity of HSCs while simultaneously impeding their ability to undergo differentiation. This phenomenon aligns with the observed functional aging of HSCs. Importantly, these changes may contribute to a predisposition to age-related diseases like myelodysplastic syndromes (MDS) and myeloid malignancies.

### 3.2. Age-Related DNA Methylation Changes and Epigenetic Clocks

Age-associated histone modification changes are accompanied by alterations in DNA methylation. Sun et al. found that in aged HSCs, DNA methylation increased at transcription factor binding sites associated with lineage potential and differentiation-promoting genes; at the same time, genes associated with HSC maintenance were relatively hypomethylated [73]. In recent years, these initial observations spurred further advances in the use of DNA methylation as a biomarker to estimate the biological age of various tissues throughout a person’s lifespan. Dubbed “epigenetic clocks,” these innovative tools employ machine learning algorithms that rely on specific CpG sites to establish a connection between the developmental and maintenance processes of biological aging. These clocks provide a means to estimate an individual’s biological age based on epigenetic modifications, contributing to our understanding of the aging process [79].

First-generation age estimators, such as Horvath’s clock [80] and Hannum’s clock [81], have found extensive use in aging and cancer research. Horvath’s clock utilizes 353 CpG sites and provides a multi-tissue and cell-type prediction of age. On the other hand, Hannum’s clock focuses on measuring the aging rate using CpG markers from whole blood. The second-generation estimators PhenoAge [82,83] and GrimAge [84] incorporated clinical phenotypes and outcomes into their models, adding further layers of complexity compared to the first-generation clocks. In a recent study, a significant breakthrough was made with the development of a single-cell age clock (scAge) [85]. This innovative approach allows the prediction of chronological age at the single-cell level in mouse tissues. This advance enhances our methodology for studying the aging process, providing a more detailed understanding of age-related changes within individual cells. These cutting-edge age estimation tools have greatly expanded our ability to study aging processes and their implications in various research areas, including age-related diseases and potential interventions [86].

## 4. Epigenetics in Acquisition of Clonal Hematopoiesis with Age

Clonal hematopoiesis (CH) is a common phenomenon associated with aging, characterized by the expansion of HSC clones, often defined by the presence of specific mutations, yet without hematologic abnormalities [87,88,89,90,91]. Epigenetic regulators *DNMT3A*, *TET2*, and *ASXL1* are the three most frequently mutated genes in CH and are also linked to the initiation of myeloid malignancies, including AML [87,91,92,93]. In healthy individuals, normal hematopoiesis is polyclonal, with peripheral blood cells originating from 50,000 to 200,000 HSCs in the bone marrow [94]. However, as individuals age, clonal hematopoiesis may develop, wherein an individual HSC clone expands and disproportionately contributes to the pool of mature blood cells [95]. The frequency of clonal events was found to increase from 0.5% in individuals younger than 35 years to over 50% in individuals older than 85 years [88]. By the age of 70, approximately 10%–20% of individuals harbor a clonal population of leukocytes in their peripheral blood, detected by the presence of somatic mutations with a variant allele fraction (VAF) of at least 2% [96,97]. Notably, there is a significant decline in clonal diversity beyond the age of 70, which is consistent across individuals [98]. The accumulation of genetic mutations during aging plays a significant role in the development of CH. Loss-of-function mutations in *DNMT3A*, *TET2*, and *ASXL1*, also known as DTA, are among the most common somatic mutations associated with CH [88] but may occasionally be germline. Unlike germline mutations, many of which are subject to elimination through purifying selection, somatic mutations tend to accumulate over the lifetime [99]. The extent to which the mutational burden is a cause or consequence of other aging processes is not fully understood [100].

While CH itself is asymptomatic, the presence of CH has important health implications in that it is associated with a significantly increased risk of all-cause mortality. In the blood system specifically, CH is associated with an 11-fold increased relative risk of a hematologic malignancy, although the absolute risk is small (5/134 vs. 11/3208 in a median follow-up period of 95 months) and varies depending on the specific gene being affected [101]. The mechanistic roles of DNMT3A and TET2 in malignant hematopoiesis have been extensively summarized elsewhere [102,103]. The impact of CH on health-related outcomes is most notable in non-hematological diseases closely associated with aging, such as cardiovascular disease (CVD) and solid tumors [87,96,100,104,105,106,107,108,109]. Among these, atherosclerosis is the most extensively studied CVD linked to CH [96,110,111,112,113,114,115,116]. The causal role of *Tet2*-driven CH in atherosclerosis has been established in *Ldlr^−/−^* mouse models engrafted with *Tet2* loss-of-function bone marrow cells; the animals exhibited accelerated development of atherosclerosis [96,115]. Additionally, in patients with no mutations in *TET2* or *DNMT3A*, a dose-dependent relationship between clone size (stratified as VAF < 1%, VAF  ≥ 1% and <2%, or VAF > 2%) and clinical outcome was observed in cardiology studies [117]. However, future clinical interpretation of CH data based on VAF should be performed with caution, as smaller clones (1–2% VAF) may also exert detrimental effects in some contexts. The interaction between CH and other CVDs has been summarized in recent reviews [95,100,101].

In patients with solid tumors, the prevalence of CH increases with age, yet it is notably more frequent than in an age-matched cancer-free population. Moreover, CH has been associated with adverse outcomes due to the progression of the primary malignancy, raising the possibility that CH may directly contribute to the development of solid tumors [101,109,118,119,120,121,122,123,124,125,126]. While the mechanism is yet to be elucidated, it is possible that CH influences cancer development through its cell–cell interactions with cancer cells, through impaired anti-tumor immune surveillance, by creating a proinflammatory milieu, or by promoting therapeutic resistance to cancer-directed treatment [125,127].

While aging leads to CH through the accumulation of mutations, CH can, in turn, accelerate aging. Recent studies have explored the relationship between CH and epigenetic aging by examining several methylation clocks, which have been shown to accurately correlate with chronological age [128,129]. Using whole-genome sequencing and methylation data from 1136 elderly individuals in the Lothian Birth Cohort, it was found that individuals with hematopoietic mutations in various types of CH drivers, including DNMT3A and TET2, had accelerated epigenetic aging as measured by the Horvath clock, a measure of intrinsic age acceleration, and other methylation clocks [129]. Similarly, in another study of 5522 individuals, it was found that CH was associated with epigenetic aging across all methylation clocks, particularly those correlating with intrinsic age acceleration [128]. When examining gene-specific associations, it was noted that mutations in *DNMT3A* and *TET2* both contributed to age acceleration, although *TET2* had significantly greater age acceleration. Moreover, individuals carrying both CH and exhibiting signs of epigenetic age acceleration were at the highest risk of all-cause mortality and coronary artery disease. Interestingly, the increased risk of CH on all-cause mortality and coronary artery disease was attenuated in individuals without age acceleration [129]. This suggests that the presence of age acceleration could be used to determine if CH carriers are at an increased risk of adverse outcomes.

**Figure 1:**
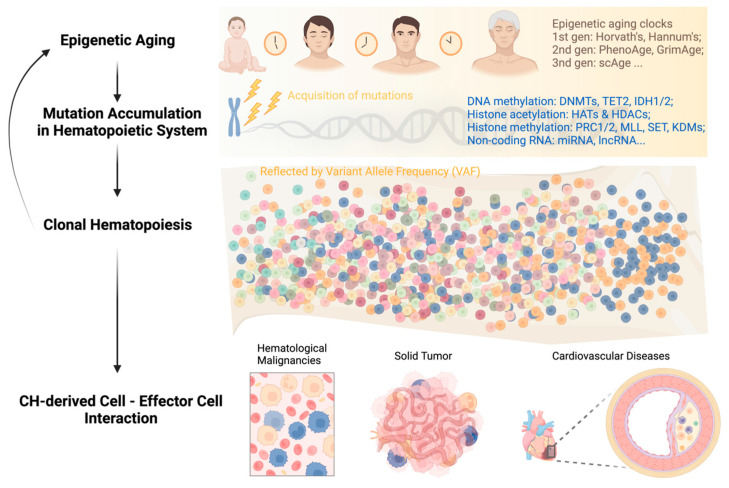
Development of clonal hematopoiesis with increasing age at its health implications.

In summary, the feedback loop of “aging—genetic mutations—clonal hematopoiesis” can persist throughout a person’s life and impact the survival of both asymptomatic individuals and patients with different types of diseases (Figure 1). Breaking this vicious circle requires multidisciplinary efforts and collaborations among researchers and clinicians in the field. Integrating CH mutation screening into clinical decision-making, including but not limited to hematological malignancies, cardiovascular disease, and solid tumors, can facilitate early diagnosis, risk stratification, and personalized disease management.

## 5. Strategies to Alleviate Aging

### 5.1. Caloric or Dietary Restriction

Caloric or dietary restriction (DR) is known to offer a plethora of health benefits, including enhanced metabolic health, neuroprotection against neurodegenerative diseases, reduced cancer incidence, and extended lifespan through intricate metabolic and epigenetic mechanisms [130,131]. However, the impact of DR on HSCs remains inconclusive. A study by Tao et al. demonstrated that early-onset DR significantly delays the aging process in HSCs, while long-term mid-onset DR improves the regenerative capacity of aging HSCs, particularly in terms of lymphoid outputs [132]. Another study by Tang et al. revealed that long-term DR, initiated from young adulthood to midlife, enhances the maintenance of HSC repopulation capacity by promoting stem cell quiescence but impairing HSC differentiation into lymphoid lineages [133]. Conversely, a study by Ho et al. indicated that although DR has been shown to delay multiple aspects of aging across various species, it does not prevent the functional decline of aged, long-term HSCs. Additionally, a long-term lifestyle intervention such as exercise and calorie restriction did not improve the function of old HSCs [134,135].

### 5.2. Small Molecule-Based Therapy

Therapeutic interventions aimed at targeting aging-related epigenetic changes hold promise for preventing or delaying the onset of hematologic disorders. By modulating epigenetic modifiers or rebalancing the cellular epigenetic landscape, it is possible to restore normal epigenetic patterns and rejuvenate aged HSCs. This approach has the potential to enhance HSC function and promote healthy hematopoiesis, opening new avenues for the treatment and prevention of hematologic disorders associated with aging.

CASIN, an inhibitor of Cdc42, has shown potential in rejuvenating old HSCs by targeting both Cdc42 activity and epigenetic reprogramming. Treatment with CASIN ex vivo has been shown to regulate Cdc42 activity and elevate H4K16Ac levels in HSCs, resembling the levels found in young cells. This treatment has demonstrated several beneficial effects, including increasing the percentage of polarized cells, restoring the spatial distribution of H4K16ac, enhancing lymphoid output, and reducing myeloid lineage output [136,137]. By reverting both the cytoskeletal polarity shift and the epigenetic landscape to a young state in HSCs, the Cdc42 inhibitor CASIN holds promise as a potential strategy for rejuvenating aged HSCs.

Histone deacetylase inhibitors (HDACis) such as valproic acid (VPA) have been used to promote retention of HSC stemness and to prevent stress-induced HSC exhaustion in early studies [138,139]. Other intervention strategies such as mTOR inhibitors (rapamycin) [140], metformin [141], resveratrol [142,143], p38 MAPK Inhibitors (TN13, SB203580) [144,145], and senolytic drugs (ABT263) [146], have also demonstrated promising results in rejuvenating aged HSCs. These interventions hold promise for mitigating age-related changes in HSC function and promoting their rejuvenation (Table 1).

### 5.3. Gene Expression Regulation

Numerous animal studies have highlighted the potential of targeting epigenetic modifiers as effective interventions. One such modifier is SIRT3, a mammalian sirtuin responsible for regulating mitochondrial acetylation. Aged mouse HSCs have been observed to exhibit reduced levels of SIRT3. However, it has been demonstrated that the overexpression of Sirt3 can rescue age-related functional defects in HSCs, ultimately restoring their long-term competitive repopulation ability [78]. Another important epigenetic modifier, SIRT7, has been found to be downregulated in aged murine HSCs. Conversely, the overexpression of *Sirt7* has shown promising results whereby it increased the reconstitution capacity of HSCs and reduced the myeloid bias typically associated with aging, thus rescuing myeloid-biased differentiation [147]. Furthermore, the overexpression of *Satb1*, an epigenetic regulator of lymphoid progenitors, has been shown to effectively rescue immunosenescence in aged HSCs. This restoration of Satb1 expression has been linked to the rejuvenation of lymphopoietic potential in aged HSCs, thereby providing a means to restore their functionality [148].

The plant homeodomain 6 (Phf6) protein plays a crucial role in chromatin organization and transcriptional regulation by associating with chromatin and interacting with the nucleosome remodeling deacetylase (NuRD) complex [149]. In a recent study, Wendorff et al. demonstrated that genetic inactivation of the *Phf6* gene mitigated age-associated HSC decline. In addition, LT-HSCs from old *Phf6*-knockout mice exhibited epigenetic rewiring and altered transcriptional programs, indicating a reduction in genotoxic stress-induced HSC aging [150].

### 5.4. Epigenetic Reprogramming

Epigenetic modifications are pivotal in governing gene expression and maintaining cellular identity. Harnessing the power of induced pluripotent stem cell (iPSC) reprogramming makes it possible to reset or remodel the aberrant epigenetic marks that accumulate during the aging process. This rejuvenation process holds immense promise for enhancing the regenerative potential of HSCs.

Wahlestedt et al. provided evidence that reprogramming aged HSCs through a pluripotent intermediate can rejuvenate their function, which was comparable to young HSCs. This suggests that alterations in the epigenome play a significant role in the aging of HSCs and contribute to the impairment of their function [151]. Recent research conducted by Yang et al. further reveals that the gradual erosion of the epigenetic landscape associated with cellular responses to double-stranded DNA breaks contributes to the acceleration of aging hallmarks. Interestingly, the aging phenotype can be reversed through epigenetic reprogramming using the overexpression of Oct4, Sox2, and Klf4 (OSK). These findings highlight the potential of epigenetic interventions in reversing the effects of aging [152].

By reversing age-related alterations in the epigenome, iPSC-based reprogramming opens new avenues for the development of innovative therapeutic approaches aimed at ameliorating age-related decline in hematopoiesis and its associated disorders.

## 6. Future Perspectives

Advances in epigenomic technologies and methodologies, including single-cell approaches and genome-editing tools, will provide deeper insights into the epigenetic regulation of hematologic aging and premalignant conditions. Exploring understudied areas, such as non-coding RNAs and three-dimensional chromatin interactions, will broaden our understanding of the complexity of epigenetic mechanisms. Additionally, combining epigenetic therapies with other treatment modalities, such as immunotherapy, may pave the way for innovative and personalized therapeutic strategies for age-related hematologic disorders.

In conclusion, this review highlights the critical role of epigenetic mechanisms in hematologic aging and premalignant conditions. Understanding the intricacies of DNA methylation, histone modifications, RNA modifications, and their interplay provides valuable insights into the underlying processes driving hematologic aging and the acquisition of clonal hematopoiesis. Targeting epigenetic dysregulation holds promise for developing novel therapeutic strategies and interventions aimed at preventing or managing age-related hematologic disorders.

## Figures and Tables

**Table 1:** Small molecule-based strategies to alleviate HSC aging.

Small Molecule	Targets	Functions
CASIN	Cdc42, elevate H4K16Ac	rejuvenate old HSCs, enhance lymphoid output, and reduce myeloid lineage output
VPA	HDACs	promote retention of HSCs stemness and prevent stress-induced HSC exhaustion
rapamycin	mTOR	restore the self-renewal and hematopoiesis of HSCs
TN13, SB203580	p38 MAPK	protect HSCs from ROS–p38 MAPK induced exhaustion, rejuvenate aged HSCs
ABT263	BCL-2 and BCL-xL	deplete senescent HSCs, attenuate HSC myeloid skewing, and improve HSC fitness
metformin	AMPK; not fully understood	improves defective hematopoiesis and reduces DNA damage
resveratrol	oxidant, sirtuins, and AMPK	increases HSC capacity and ameliorates irradiation-induced HSC injury

## Data Availability

Not applicable.
